# Colorectal Cancer and Precursor Lesion Prevalence in Adults Younger Than 50 Years Without Symptoms

**DOI:** 10.1001/jamanetworkopen.2023.34757

**Published:** 2023-12-06

**Authors:** Daniela Penz, Elisabeth Waldmann, Monika Hackl, Lena Jiricka, Lisa-Maria Rockenbauer, Irina Gessl, Jasmin Zessner-Spitzenberg, Arnulf Ferlitsch, Michael Trauner, Monika Ferlitsch

**Affiliations:** 1 Quality Certificate for Screening Colonoscopy, Austrian Society of Gastroenterology and Hepatology, Vienna, Austria; 2 Division of Gastroenterology and Hepatology, Department of Internal Medicine III, Medical University of Vienna, Vienna, Austria; 3 Department of Internal Medicine I, St John of God Hospital, Vienna, Austria; 4 Statistic Austria, Vienna, Austria; 5 Department of Bioimetrics, Medical University of Vienna, Vienna, Austria

## Abstract

**Importance:**

Incidence of colorectal cancer (CRC) is increasing among younger adults. However, data on precursor lesions in patients who are asymptomatic, especially those aged younger than 50 years, are lacking.

**Objective:**

To analyze the prevalence and number needed to screen (NNS) for adenomas, advanced adenomas, and serrated lesions, as well as the incidence of CRC in patients older than age 20 years.

**Design, Setting, and Participants:**

This cohort study was conducted among 296 170 patients who received a screening colonoscopy within a national screening colonoscopy registry from 2012 to 2018 in Austria, including 11 103 patients aged younger than 50 years. CRC incidence was analyzed using data from Statistic Austria from 1988 to 2018. Data were analyzed in September 2021.

**Main Outcome and Measures:**

The prevalence of adenomas and other lesions and the incidence of CRC in individuals aged 20 years or older were assessed.

**Results:**

Among 296 170 patients included in the study (median [IQR] age, 60 [54-68] years; 150 813 females [50.9%]), 11 103 patients (3.7%) were aged younger than 50 years and 285 067 patients (96.3%) were aged 50 years or older. Among patients younger than age 50 years, 1166 individuals (10.5%; NNS = 9) had adenomas and 389 individuals (3.9%; NNS = 26) had at least 1 advanced adenoma, while among those aged 50 years or older, 62 384 individuals (21.9%; NNS = 5) had adenomas and 19 680 individuals (6.9%; NNS = 15) had at least 1 advanced adenoma. Among 1128 males aged 40 to 44 years, 160 individuals (14.2%; NNS = 7) had at least 1 adenoma, and among 1398 females aged 40 to 44 years, 114 individuals (8.1%; NNS = 12) had at least 1 adenoma. The prevalence of adenomas for individuals aged 45 to 49 years vs 50 to 54 years was 490 of 2879 males (17.1%; NNS = 6) vs 8269 of 40 935 males (20.2%; NNS = 5) and 284 of 2792 females (10.2%; NNS = 10) vs 4997 of 40 303 females (12.4%; NNS = 8), respectively. Prevalence of adenomas changed from 61 of 498 individuals (12.4%) in 2008 to 150 of 1064 individuals (14.1%) in 2018 among those younger than 50 years and from 2646 of 12 166 individuals (21.8%) to 10 673 of 37 922 individuals (28.2%) among those aged 50 years and older. The prevalence of advanced adenomas changed from 20 individuals (4.0%) in 2008 to 55 individuals (5.2%) in 2018 in individuals younger than 50 years and from 888 individuals (7.3%) in 2008 to 2578 individuals (6.8%) in 2018 among those aged 50 years and older. Among individuals younger than age 50 years, CRC incidence per 100 000 individuals changed from 9.1 incidents in 1988 to 10.2 incidents in 2018 among males (average annual percentage change [AAPC], 0.5%; 95% CI, 0.1% to 1.0%) and from 9.7 incidents in 1988 to 7.7 incidents in 2018 among females, with a nonsignificant AAPC (−0.2%; 95% CI, −0.7% to 0.3%). Among individuals aged 50 years or older, CRC incidence per 100 000 individuals changed from 168 incidents in 1988 to 97 incidents in 2018 among females (AAPC, −1.8%; 95% CI, −1.9% to −1.6%), and 217 incidents in 1988 to 143 incidents in 2018 among males (AAPC, −1.2%; 95% CI, −1.3% to −1.1%).

**Conclusion:**

In this study, CRC incidence decreased after 1988 in Austria among individuals older than 50 years, while among patients younger than 50 years, incidence increased among males but decreased among females. Prevalence of adenomas increased in all age groups, while advanced adenoma prevalence increased among patients younger than 50 years but decreased in patients aged 50 years and older.

## Key Points

**Question:**

What is the trend in colorectal cancer (CRC) incidence among younger adults in Austria and what are the trends for adenomas, advanced adenomas, and serrated lesions in adults younger than age 50 years?

**Findings:**

In this cohort study of 296 170 patients who received a screening colonoscopy, CRC incidence decreased in Austria in those aged 50 years or older but increased among males aged younger than 50 years and decreased in females aged younger than 50 years. Adenoma prevalence increased across age groups, while advanced adenoma prevalence increased in patients aged younger than 50 years but decreased in those aged 50 years or older.

**Meaning:**

This study found increasing incidence of CRC in younger adults, suggesting the importance of targeted screening efforts, especially for males aged younger than 50 years.

## Teaser

This cohort study investigates the incidence of colorectal cancer and prevalence of adenomas and other precursor lesions among adults younger than 50 years in Austria.

## Introduction

Colorectal cancer (CRC) incidence and mortality decreased in the US and many European countries over the last 30 years among adults aged 55 years and older, which could be an effect of higher adherence to CRC screening programs. At the same time, an increase in mortality and incidence rates among younger patients was noticed.^1^

Given that the incidence of CRC increases around age 50 years within a mean-risk population, screening programs have focused on this population so far. The American Cancer Society recommends that the age for individuals at mean risk undergoing first-time screening colonoscopy should be lowered to 45 years.^2^

With the purpose of detecting and removing precancerous lesions, such as adenomas, within the same investigation, colonoscopy is the criterion standard for CRC screening. While having a higher risk of progression to CRC, adenomas are divided into nonadvanced (tubular adenomas <10 mm) adenomas and advanced (≥10 mm in size, high-grade dysplasia, or villous histology) adenomas (AAs), and transition rates from AA to CRC were reported to greatly increase with age but were similar between male and female patients.^3^

In addition to age, patient sex should also have an influence on the age at which a screening colonoscopy is recommended given that several studies have found that male sex is an independent factor associated with CRC.^4^ In a study by Ferlitsch et al,^5^ the incidence of CRC and precursors in males aged 45 to 49 years was comparable with that of females 10 years older.

Meester et al^6^ showed that CRC among individuals aged 40 to 45 years was diagnosed at a more advanced stage compared with in 1990. This indicates a real increase of CRC incidence and not only a shift in age at diagnosis. Despite the evidence of an increase in CRC incidence among younger adults,^7,8^ data about the prevalence of precursor lesions, such as adenomas, among younger individuals, especially those without symptoms, are still missing.

Therefore, the aim of our study was to assess the prevalence of adenomas and AAs among younger adults without symptoms within the Austrian CRC screening program. Furthermore, the incidence of CRC within younger adults was evaluated and trends since 1988 were analyzed using data from Statistic Austria.

## Methods

This cohort study was approved by the local ethics committee of the Medical University of Vienna. All patients had to sign written informed consent giving permission for data submission. The study was conducted following the Strengthening the Reporting of Observational Studies in Epidemiology (STROBE) reporting guideline.

### Study Design

There were 305 229 screening colonoscopies analyzed within the Austrian quality assurance program for screening colonoscopies, which was established in 2007 by the Austrian Society of Gastroenterology and Hepatology together with the National Cancer Aid. Participating endoscopists are surgeons, specialists for internal medicine, or gastroenterologists who must provide proof of at least 200 colonoscopies and 50 polypectomies under supervision and 100 unsupervised performed colonoscopies and 10 polypectomies per year. Approximately 45% of endoscopists in Austria participate in this project. Participating centers and physicians collect colonoscopy reports and histopathological analyses, which are transferred electronically to our database using a web form. An annual random sample checkup is conducted to check correctness of submitted data. Additionally, photo documentation is necessary to assess if cecum was reached. Twice a year, participants receive benchmark reports to inform them about their quality measures, such as adenoma detection rate, cecal intubation rate, sedation rate, and further parameters, to get feedback on their performance. Information regarding serrated lesions (sessile serrated lesions and traditional serrated adenomas) was added to the report form in 2012. In 236 of 463 endoscopists participating in the quality assurance program (51.0%), participating physicians were using high-definition endoscopes. Further descriptions of this project have been reported elsewhere.^9,10,11^

Data were provided from 305 229 screening colonoscopies between 2008 and 2018, mostly involving patients aged 50 years and older. However, younger individuals were allowed to undergo a screening colonoscopy within this program if they were asymptomatic and the reason for the examination was patient fear of cancer. Patients with a positive family history, inflammatory bowel disease (IBD), or cancer symptoms were excluded from this study. Information regarding CRC incidence in Austria was provided by Statistic Austria for 1988 until 2018.

### Exclusion and Inclusion Criteria

Endoscopists with fewer than 30 screening colonoscopies (560 individuals) and examinations with poor bowel preparation (8499 examinations) were excluded from this study, resulting in 9059 exclusions. A total of 296 170 screening colonoscopies remained after exclusion for study analyses within the screening program.

### Statistical Analysis

Patient characteristics were described using median and IQR and categorical variables by absolute and relative frequencies. We used the χ^2^ test to evaluate differences between groups. We divided all patients into age groups by steps of 5 years and calculated the prevalence of adenomas and AAs and the number needed to screen (NNS) for each group. AAs were defined as adenomas 10 mm or greater in size, with high-grade dysplasia, or with villous histology. Prevalences over time for adenomas and AAs were analyzed from 2008 to 2018. Information regarding serrated lesions, including sessile serrated lesions (SSLs) and traditional serrated adenomas (TSAs) were added to the colonoscopy report in 2012. Prevalence of serrated lesions was analyzed between 2012 and 2018. Austrian data on CRC incidence from 1988 to 2918 were provided by Statistics Austria. Piecewise linear regression models were fitted using the segmented R package version 1.2. The number of breakpoints was limited to 1. We used the average annual percentage change (AAPC) to analyze trends in the incidence of CRC and precursor lesions. Statistical significance was denoted as *P* ≤ .05. Analyses were conducted as 2-sided tests using R statistical software version 4.1.0 (R Project for Statistical Computing). Data were analyzed from January 2012 to December 2018.

## Results

A total of 296 170 patients underwent a screening colonoscopy within the Austrian quality assurance program between 2008 and 2018 and were included in this study (150 813 females [50.9%] and 145 357 males [49.1%]; median [IQR] age, 60 [54-68] years). There were 11 103 patients (3.8%) aged younger than 50 years (median [IQR] age, [40-48] years) and 285 067 patients (96.3%) aged 50 years or older (median [IQR] age, 60 [54-68] years) (Table 1).

**Table 1.** Patient Baseline Characteristics

Characteristic	Patients, No. (%)		
	Overall (N = 296 170)	Age <50 y (n = 11 103)	Age ≥ 50 y (n = 285 067)
Sex			
Female	150 813 (50.9)	5744 (51.7)	145 069 (50.8)
Male	145 357 (49.1)	5359 (48.3)	139 998 (49.2)
Age, median (IQR)	60 (54-68)	45 (40-48)	60 (54-68)

### Quality Parameters

Cecum was reached in 287 585 patients (97.1%). In 640 patients (0.2%), complications were registered, including 42 perforations (<0.1%). The median (IQR) detection rate was 21.4% (17.6-26.1%) for adenomas and 6.6% (4.2%-9.0%) for AAs

### CRC Incidence

Data from Statistic Austria showed that CRC incidence per 100 000 individuals among males younger than 50 years changed from 9.1 incidents in 1988 to 8.5 incidents in 1998, 8.6 incidents in 2008, and 10.2 incidents in 2018 (AAPC, 0.5%; 95% CI, 0.1% to 1.0%). Incidence per 100 000 individuals among females in the same age group was 9.7 incidents in 1988, 7.0 incidents in 1998, 7.8 incidents in 2008, and 7.7 incidents in 2018, with a nonsignificant AAPC (−0.2%; 95% CI, −0.7% to 0.3%). Among males older than 50 years, rates per 100 000 individuals changed from 217 incidents in 1988 to 220 incidents in 1998, 199 incidents in 2008, and 143 incidents in 2018 (AAPC, −1.2%; 95% CI, −1.3% to −1.1%). Incidence per 100 000 female patients aged 50 years or older was 168 incidents in 1988, 166 incidents in 1998, 132 incidents in 2008, and 97 incidents in 2018 (AAPC, −1.8%; 95% CI, −1.9% to −1.6%)

**Figure 1.**
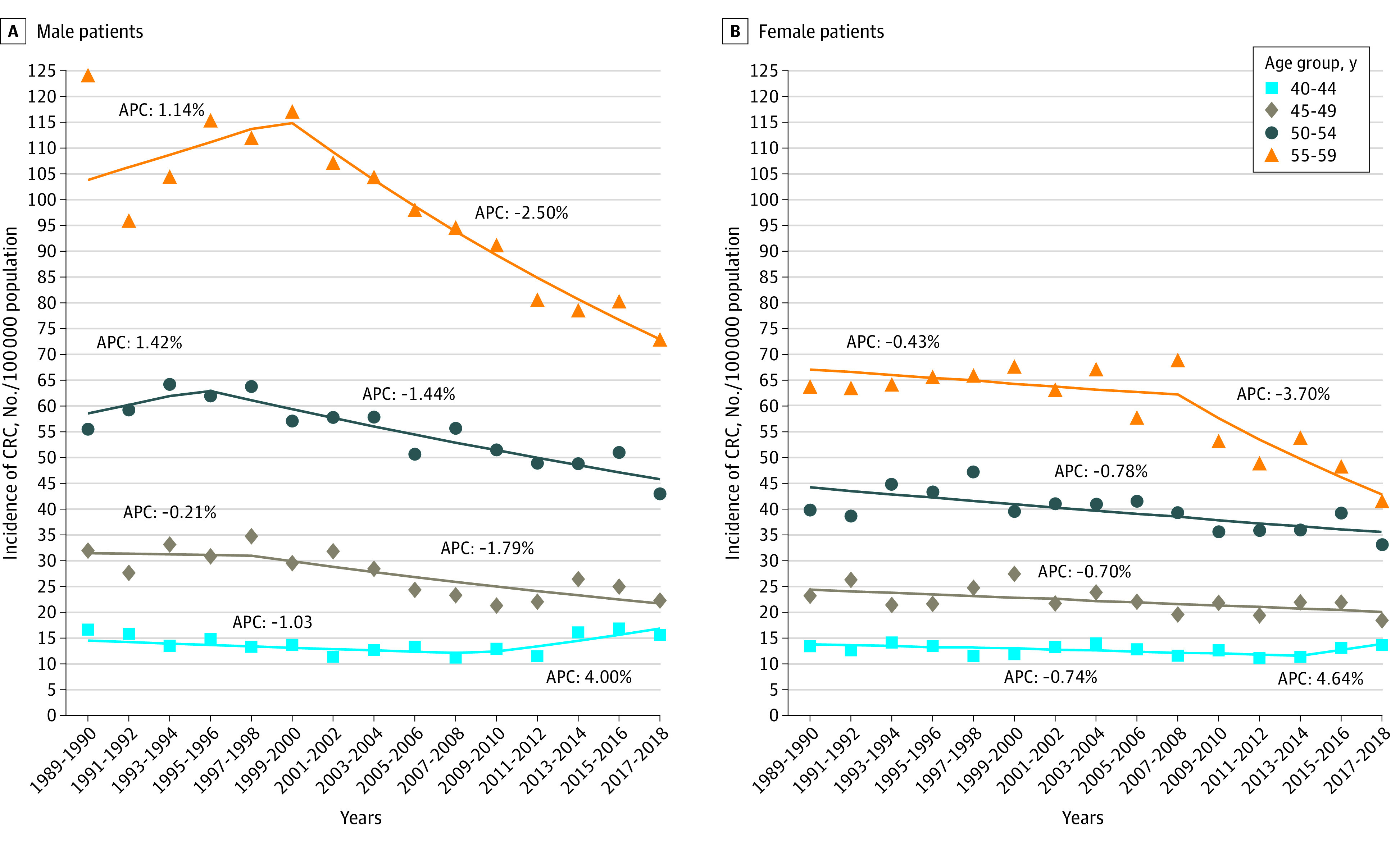
Incidence of Colorectal Cancer (CRC) Incidence and estimated annual percentage change (APC) of CRC are given by age and sex.

CRC incidence per 100 000 individuals among males aged 40 to 44 years changed from 14.4 incidents in 1988 to 15.1 incidents in 1998, 12.5 incidents in 2008, and 16.2 incidents in 2018, for a nonsignificant AAPC (0.4%; 95% CI, −0.4% to 1.1%). In females in this age group, incidence per 100 000 individuals was 11.7 incidents in 1988, 10.2 incidents in 1998, 11.9 incidents in 2008, and 16.5 incidents in 2018, for a nonsignificant AAPC (0.1%; 95% CI, −0.8% to 1.0%). Among individuals aged 45 to 49 years, the incidence per 100 000 individuals changed from 92 incidents in 1988 to 77 incidents in 2018 among males (AAPC, −1.1%; 95% CI, −1.7% to −0.5%) and from 84 incidents in 1988 to 53 incidents in 2018 among females, for a nonsignificant AAPC (−0.9%; 95% CI, −0.8% to 1.0%). Among males aged 50 to 54 years, incidence of CRC per 100 000 individuals changed from 112 incidents in 1988 to 155 incidents in 2018 (AAPC, −0.7%; 95% CI, −1.0% to −0.4%). Among females, incidence per 100 000 individuals was 95 incidents in 1985 and 134 incidents in 2015 (AAPC, −0.5%; 95% CI, −1.0 to −0.7). Among individuals aged 55 to 59 years, incidence per 100 000 individuals changed from 213 incidents in 1988 to 223 incidents in 2018 (AAPC, −1.2%; 95% CI, −1.5% to −0.9%) among males and from 139 incidents in 1988 to 135 incidents in 2018 (AAPC, −1.3%; 95% CI, −1.6% to −1.0%) among females (Figure 1).

### Prevalence, AAPC, and NNS of Adenomas

**Figure 2.**
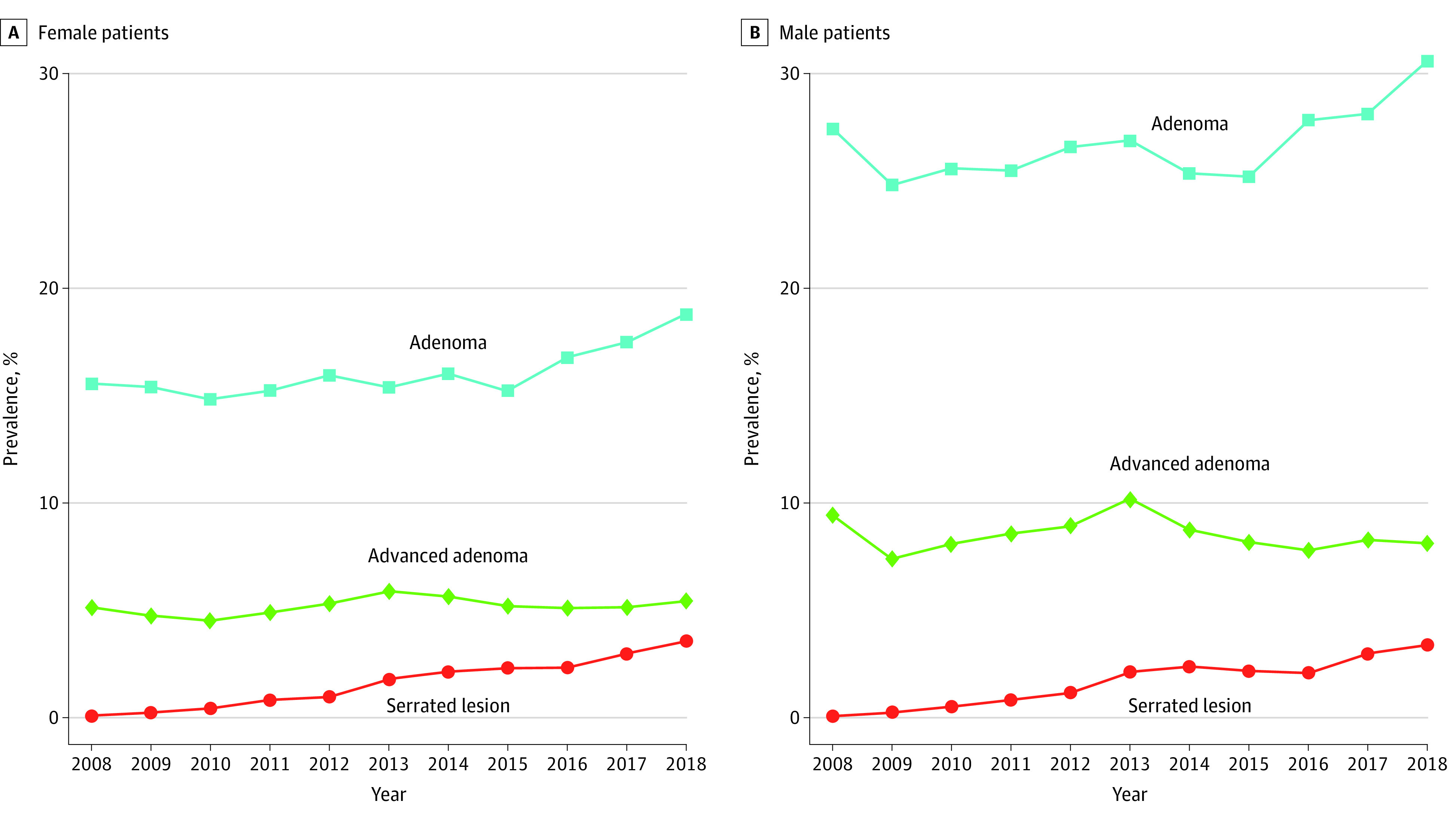
Prevalence of Adenomas by Sex The prevalence of adenomas is given for males and females.

**Figure 3.**
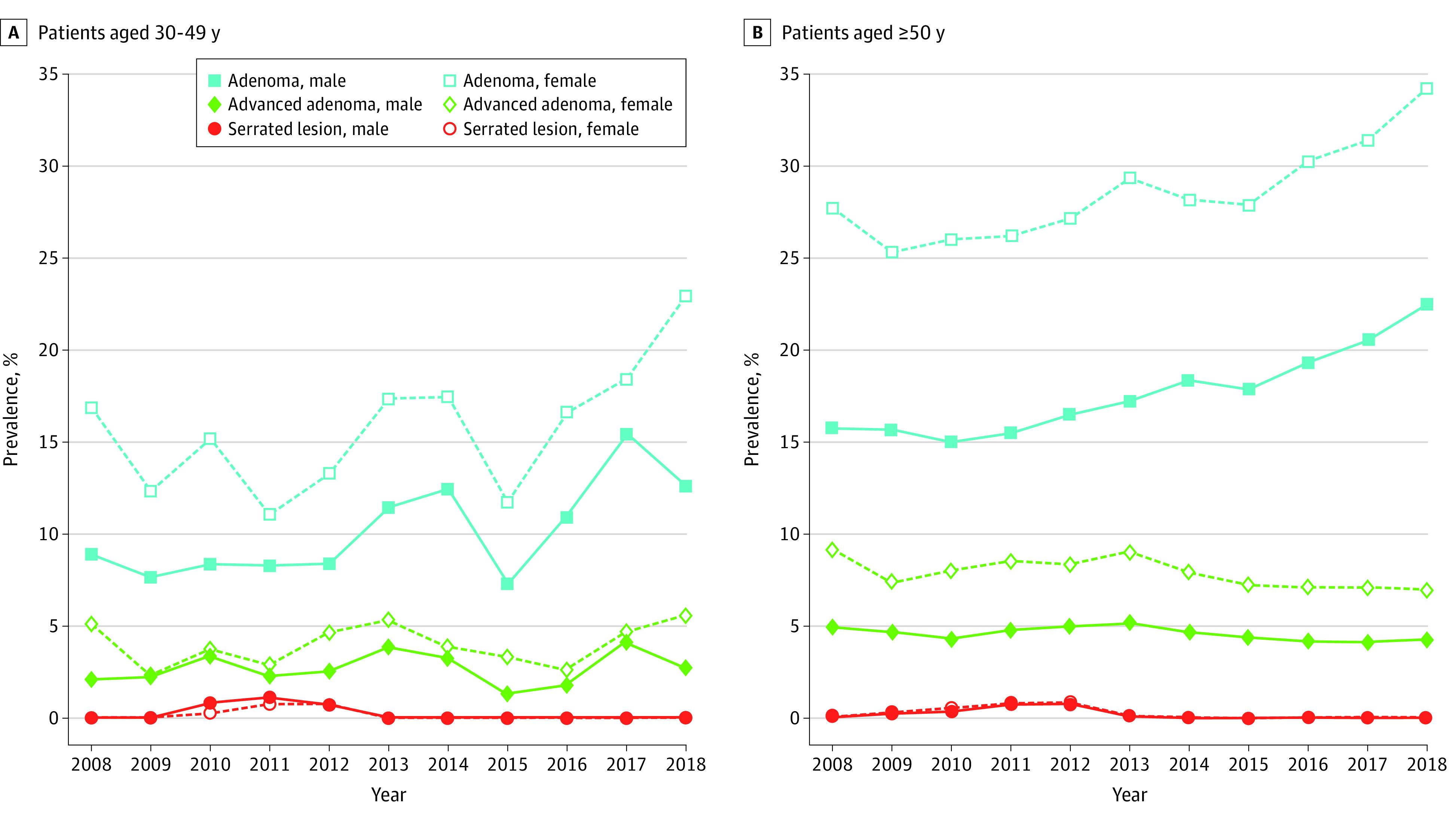
Prevalences of Adenomas, Advanced Adenomas, and Serrated Lesions Prevalences of adenomas, advanced adenomas, and serrated lesions are given per year.

Among all patients, 63 550 individuals (21.4%; NNS = 5) had at least 1 adenoma; 39 094 males (26.9%; NNS = 4) and 24 456 females (16.2%; NNS = 6) had adenomas (*P* < .001). Among patients younger than age 50 years, 1166 individuals (10.5%; NNS = 9) had adenomas, whereas among patients aged 50 years or older, 62 384 individuals (21.9%; NNS = 5) had adenomas (*P* < .001). Among 5359 males younger than age 50 years, 710 individuals (13.2%; NNS = 7) had adenomas, and among 5744 females younger than age 50 years, 456 individuals (7.9%; NNS = 12) had adenomas. In patients aged 50 years or older, 62 384 individuals overall (21.9%;NNS = 5) had adenomas, while 38 384 of 139 998 males (27.4%; NNS = 4) and 24 000 of 145 069 females (16.5%; NNS = 6) had adenomas. Among individuals younger than age 50 years, the prevalence changed from 61 of 498 individuals (12.4%) in 2008 to 150 of 1064 individuals (14.1%) in 2018 overall, 38 of 226 males (16.8%) in 2008 to 97 of 512 males (19.0%) in 2018, for a nonsignificant AAPC (2.6%; 95% CI, −1.2% to 6.5%), and 23 of 272 females (8.5%) in 2008 to 53 of 552 females (9.7%) in 2018, for a nonsignificant AAPC (3.1%; 95% CI, −1.8% to 8.1%). Among individuals aged 50 years or older, the prevalence changed from 2646 of 12 166 individuals (21.8%) in 2008 to 10 673 of 37 922 individuals (28.2%) in 2018 overall, 1626 of 5840 males (27.8%) in 2008 to 6313 of 18 461 males (34.2%) in 2018 (AAPC, 1.4%; 95% CI, 0.7% to 2.1%), and 1020 of 6326 females (16.1%) in 2008 to 4360 of 19 461 females (22.4%) in 2018 (AAPC, 2.0%; 95% CI, 1.4% to 2.5%) (Figure 2 and Figure 3).

Among 2526 individuals aged 40 to 44 years, adenomas were found in 274 individuals (10.9%; NNS = 9) overall, 114 of 1398 females (8.1%; NNS = 12), and 160 of 1128 males (14.2%; NNS = 7) (Table 2). Within this age group, the prevalence of adenomas changed from 11 of 69 males (15.9%) in 2008 to 9 of 44 males (20.4%) in 2018, for a nonsignificant AAPC (3.6%; 95% CI, −3.0% to 10.7%), and 4 of 52 females (7.7%) in 2008 to 4 of 109 females (3.7%) in 2018 (AAPC, −1.5%; 95% CI, −1.5% to −1.5%) in 2018.

**Table 2.** Prevalence of Adenomas and AAs

Age group, y	Sex	Total patients, No.	Prevalence, No. (%) [NNS]		
			Adenoma	AA	Serrated lesion
40-44	Both	2526	274 (10.9) [9.2]	98 (3.9) [26.7]	38 (1.5) [66.7]
	Male	1128	160 (14.2) [7.1]	66 (5.9) [16.9]	19 (1.7) [58.8]
	Female	1398	114 (8.1) [12.3]	32 (2.3) [43.5]	19 (1.4) [71.4]
45-49	Both	5671	774 (13.6) [7.4]	256 (4.5) [22.2]	110 (1.9) [52.6]
	Male	2879	490 (17.1) [65.9]	144 (5.0) [20.0]	52 (1.8) [55.5]
	Female	2792	284 (10.2) [10.2]	112 (4.0) [25.0]	58 (2.1) [47.6]
50-54	Both	81 238	13 266 (16.3) [6.1]	3825 (4.7) [21.3]	1462 (1.8) [55.5]
	Male	40 935	8269 (20.2) [5.0]	2374 (5.8) [17.2]	777 (1.8) [55.5]
	Female	40 303	4997 (12.4) [8.4]	1451 (3.6) [27.8]	685 (1.7) [58.8]
55-59	Both	58 370	11 732 (20.1) [4.0]	3561 (6.1) [16.4]	1130 (1.9) [52.6]
	Male	28 540	7302 (25.6) [3.9]	2169 (7.6) [13.2]	540 (1.9) [52.6]
	Female	29 830	4430 (14.9) [6.7]	1392 (4.7) [21.3]	590 (2.0) [50.0]
60-64	Both	49 417	11668 (23.6) [4.2]	3710 (7.5) [13.3]	935 (1.9) [52.6]
	Male	23 919	7104 (29.7) [3.4]	2310 (9.7) [10.3]	461 (1.9) [52.6]
	Female	25 498	4564 (17.9) [5.6]	1400 (5.5) [18.2]	474 (1.9) [52.6]

Abbreviations: AA, advanced adenoma; NNS, number needed to screen.

Among 5671 individuals aged 45 to 49 years, 774 individuals overall (13.6%; NNS = 7), 490 of 2879 males (17.1%; NNS = 6), and 284 of 2792 females (10.2%; NNS = 10) had adenomas (Table 2). Among males in this age group, the prevalence of adenomas changed from 22 of 109 males (20.2%) in 2008 to 56 of 273 males (20.5%) in 2018 (AAPC, 0.4%; 95% CI, 3.4% to 2.7%) and from 16 of 135 females (11.2%) in 2008 to 34 of 289 females (11.8%) in 2018, for a nonsignificant AAPC (2.6%; 95% CI, −2.7% to 8.1%).

Among 81 238 individuals aged 50 to 54 years, 8269 of 40 935 males (20.2%; NNS = 5) and 4997 of 40 303 females (12.4%; NNS = 8) had adenomas (Table 2). In this age group, there was an increase from 301 of 1455 males (20.7%) in 2008 to 1330 of 5407 males (24.7%) in 2018 (AAPC, 1.2%; 95% CI, 0.5%-1.8%) and 175 of 1501 females (11.7%) in 2008 to 800 of 5441 females (14.7%) in 2018 (AAPC, 2.5%; 95% CI, 1.9%-3.1%). Among 58 370 patients aged 55 to 59 years, prevalence changed from 316 of 1266 males (25.0%) in 2008 to 1189 of 3825 males (31.1%) in 2018 and 181 of 1370 females (13.2%) in 2008 to 678 of 4016 females (16.9%) in 2018.

### Prevalence and NNS of AAs

AAs were detected in 20 069 patients overall (6.8%; NNS = 15), 12 271 males (8.5%; NNS = 12), and 7798 females (5.2%; NNS = 19) (*P* < .001). Among individuals younger than age 50 years, 389 individuals overall (3.9%; NNS = 26), 229 males (4.6%; NNS = 22) and 160 females (3.1%; NNS = 32) had AAs. Among individuals aged 50 years or older, 19 680 individuals overall (6.9%; NNS = 15), 12 042 males (8.6%; NNS = 12), and 7638 females (5.3%; NNS = 19) had at least 1 AA. The proportion of patients with an AA detected differed statistically significantly by age group (<50 vs ≥50 years: *P* < .001).

The prevalence of AAs among all patients aged younger than 50 years changed from 20 individuals (4.0%) in 2008 to 55 individuals (5.2%) in 2018 overall, 21 males (9.3%) in 2008 to 42 males (8.2%) in 2018, and 7 females (2.5%) in 2008 to 2 females (3.9%) in 2018. Among patients aged 50 years and older, the prevalence changed from 888 individuals (7.3%) in 2008 to 2578 individuals (6.8%) in 2018 overall, 329 females (5.2%) in 2008 to 817 females (4.2%) in 2018, and 555 males (9.5%) in 2008 to 1514 males (8.2%) in 2018 (Figure 3).

Among patients aged 40 to 44 years, AAs occurred in 98 individuals overall (3.9%; NNS = 27), 66 males (5.9%; NNS = 17), and 32 females (2.3%; NNS = 44). Within the age 45 to 49 years group, AAs occurred in 144 males (5.0%; NNS = 20) and 112 females (4.0%; NNS = 25). In this age group, AAs occurred in 6 males (5.5%) in 2008 and 11 males (4.0%) in 2018 and 4 females (3.0%) in 2008 and 9 females (3.1%) in 2018.

In patients aged 50 to 54 years, 2374 males (5.8%; NNS = 17) and 1451 females (3.6%; NNS = 28) had AAs. Within this age group, 77 males (5.3%) had at least 1 AA in 2008 compared with 249 males (4.6%) in 2018, while 45 females (3.0%) in 2008 and 169 females (3.1%) in 2018 had at least 1 AA. Among patients aged 55 to 59 years, the prevalence changed from 96 males (7.6%) in 2008 to 241 males (6.3%) in 2018 and 59 females (4.3%) in 2008 to 120 females (3.0%) in 2018.

### Prevalence and Trends of Serrated Lesions

Serrated polyps, including SSLs and TSAs, were detected in 5410 individuals (1.8%) overall, 165 patients aged younger than 50 years (1.6%), and 5245 patients aged 50 years or older (1.8%). Among patients younger than age 50 years, 79 males (1.6%) and 86 females (1.7%) had serrated polyps, while among those aged 50 years or older, serrated lesions were detected in 2578 males (1.8%) and 2667 females (1.8%).

Within the ages 45 to 49 years group, 110 individuals overall (1.9%), 58 females (2.1%), and 52 males (1.8%) had serrated lesions. Among individuals aged 50 to 54 years, 1462 individuals overall (1.8%), 685 females (1.7%), and 777 males (1.8%) had serrated lesions.

From 2012 to 2018, the prevalence of serrated lesions changed from 241 of 24 109 patients (1.0%) to 1325 patients (3.4%) overall, 114 of 12 653 females (0.9%) to 700 females (3.5%), and 114 of 11 456 males (1.0%) to 626 males (3.3%). Among patients younger than age 50 years, prevalence from 2012 to 2018 changed from 10 of 861 patients (1.2%) to 26 patients (3.0%) overall, 5 of 409 males (1.2%) to 21 males (4.1%), and 5 of 452 females (0.9%) to 17 females (3.1%). Among individuals aged 50 years or older, the prevalence from 2012 to 2018 changed from 232 of 23 248 individuals (1.0%) to 1289 individuals (3.4%), 110 of 11 047 males (1.0%) to 609 males (3.3%), and 110 of 12 201 females (0.9%) to 681 females (3.5%) (Figure 3).

## Discussion

This large cohort study of 296 170 screening colonoscopies among patients who were asymptomatic, including 11 103 patients (3.7%) younger than age 50 years, found an increased prevalence of adenomas among younger adults since 2008. However, this trend was seen in all age groups. Therefore, these findings may not necessarily reflect a general increase in the presence of adenomas within younger adults but rather an improvement in screening quality and adenoma detection rate in general, as well as better equipment, which may be associated with higher numbers of detected adenomas. A study by Ahn et al^12^ found that the miss rate for adenomas larger than 10 mm was 5.8%, showing that high-risk lesions have a relatively small risk for not being detected during colonoscopy. In our study cohort, we additionally found that the prevalence of AAs and the incidence of CRC were increasing, which cannot be explained only by quality improvement of screening colonoscopy. However, the prevalence of AAs, which have a higher risk of progression to CRC, changed from 4.0% in 2008 to 5.2% in 2018 among patients younger than age 50 years, while it decreased from 7.3% to 6.8% in patients aged 50 years or older.

Despite the increase in adenomas, the incidence of CRC in Austria decreased from 1988 to 2018 in males and females aged 50 years and older. This is likely associated with trends of the opportunistic screening program in Austria. The incidence in males younger than age 50 years increased. The incidence per 100 000 individuals of CRC in females younger than age 50 years changed from 9.7 incidents in 1988 to 7.7 incidents in 2018. However, the AAPC within this group was −0.2%, which suggests that the incidence in this group decreased when every year of this period is statistically taken into account.

Looking at different age groups, an increase of CRC incidence was noticed within males aged 40 to 44 years, while the AAPC within males aged 45 to 49 years declined. Within females aged 40 to 44 years, we also found an increase in CRC incidence, although the AAPC of 0.1% was not statistically significant, while the incidence within females aged 45 to 49 years decreased, although the AAPC of −0.9% was not statistically significant. In Austria, the age to start with screening colonoscopy was 50 years during the period analyzed in this study. Nevertheless, an increase in CRC incidence in younger adults was greater within males aged 40 to 44 years.

However, in 10.5% of patients younger than age 50 years, adenomas were found. The NNS of 9 represents that 9 patients younger than age 50 years need to be screened to find 1 adenoma. The NNS in those aged 45 to 49 years was almost comparable with that of those aged 50 to 54 years in this study.

A study by Ferlitsch et al^5^ showed that the prevalence and NNS of AA in women were comparable to those in women aged 10 years younger. Our study found that the prevalence was higher and the NNS lower within males aged 40 to 44 years (5.9%; NNS = 18) than that among males aged 45 to 49 years (5.0%; NNS = 20). Thus, the NNS within males aged 40 to 44 years was comparable to that in females aged 20 years older (aged 60-64), with a prevalence of 5.5% and an NNS of 18.

Some studies reported an increase of CRC in younger adults. In a study by Vuik et al,^13^ CRC incidence increased within individuals aged 20 to 49 years, especially those aged 20 to 39 years. However, the reason for undergoing screening colonoscopy within those patients is unknown, and the trend of increasing incidence may be due to a selection bias. Furthermore, no increase in mortality was found. A study by Sehgal et al^14^ showed that a colonoscopy was associated with a decrease in the risk of CRC by 50% when given at ages 45 to 49 years and of 68% when given at ages 50 to 54 years. However, most study patients were referred to colonoscopy due to symptoms, so outcomes associated with lowering the age to start screening colonoscopy within this age group remain unclear.

To our knowledge, our study is the first to evaluate trends in the incidence of CRC and precursor lesions within a large cohort of younger adults without symptoms, with a total of 11 103 screening colonoscopies in patients younger than age 50 years. Patients with IBD, cancer or CRC symptoms, or a positive family history of CRC were excluded from the study. The main reason for screening within those patients was patient fear of cancer. Additionally, the reason for the increasing prevalence of precursor lesions among younger adults is still unknown. Lack of physical activity, obesity, and other lifestyle factors are associated with an increased risk of CRC and adenomas.^15^ However, a limitation of our study was that we did not have data regarding lifestyle factors or comorbidities.

Moreover, our data also showed an increased incidence of serrated lesions, including SSLs and TSAs, from 2012 to 2018 in individuals aged younger than 50 years and 50 years or older. However, serrated legions were found in a total of 165 patients younger than age 50 years, not allowing for conclusions regarding the AAPC in this group. Starting in 2012, we have included sessile and traditionally serrated lesions in our report forms. Through our regular benchmark distributions and educational sessions, there has been an increasing emphasis on serrated lesions, which may account for the observed increase over the years.

The prevalence for adenomas in males aged 40 to 44 years in this study was 14.0%, suggesting that a screening colonoscopy finds an adenoma in every seventh male patient. Individuals aged 45 to 49 years already have access to screening colonoscopy in many countries. However, the NNS of 6 within males in this group is almost comparable with that of males aged 40 to 44 years, but those individuals do not have easy access to screening. The prevalence of AAs in males was higher within those aged 40 to 44 years than those aged 45 to 49 years, which raises the question of whether we should start at age 40 years, especially in males. Our data show that incidence within males increased more than that in females and the sex-specific gap is now even higher. These findings suggest that not only age but also sex should be considered for further recommendations. Our findings suggest that screening at age 40 years for males and age 50 years for females warrants thoughtful consideration.

Despite increasing incidence of adenomas and AAs among younger adults, the incidence of CRC increased only slightly among younger adults in general, which was mostly due to increasing incidence within males. In a 2022 study by Bretthauer et al,^16^ screening colonoscopy reduced the risk of CRC, but there was no effect on CRC mortality. One reason could be that it takes more time to see the effects of screening on CRC mortality. However, that study included only patients older than age 50 years, and studies evaluating the effect of screening colonoscopy on CRC incidence and mortality in younger adults are still lacking.

Given that a screening colonoscopy is a procedure that depends on operator performance, quality improvement of screening should continue. Organized screening programs should be established in all countries with the aim of offering high-quality screening endoscopy and preventing progression to colorectal cancer.

### Limitations

Our study has several limitations, including that it is primarily a descriptive study. Furthermore, this study did not analyze factors associated with CRC. We lacked data regarding comorbidities, such as smoking, diabetes, fatty liver disease, or body mass index. Therefore, further studies are necessary to evaluate the reasons for increasing incidences of CRC in younger adults, particularly males. Additionally, serrated lesions were added to our report form in 2012, so data on these types of lesions from 2008 to 2012 are missing. Given that the total number of SSLs and TSAs was quite low, another limitation of this study is that we did not have the AAPC of the prevalence of serrated lesions.

## Conclusion

This cohort study found that the prevalence of adenomas and AAs increased among younger adults in Austria. The NNS of 18 for the prevalence of AAs in males aged 40 to 44 years was lower than in males aged 45 to 49 years (NNS = 20). CRC incidence has increased since 1988 in males but not females younger than age 50 years. These findings suggest that patient sex should be considered as a factor when determining the age for starting screening in further recommendations. Based on this study, screening should have started at age 40 years for males and age 50 years or even later, around age 55 years, for females.

## Notes

Supplement.
Data Sharing Statement
