# Supplementary material for: Colorectal Cancer and Precursor Lesion Prevalence in Adults Younger Than 50 Years Without Symptoms
Source: JAMA Netw Open. 2023 Dec 6;6(12):e2334757. doi: 10.1001/jamanetworkopen.2023.34757 (PMC10701609; doi:10.1001/jamanetworkopen.2023.34757)
Supplement: Supplement. — Data Sharing Statement [file jamanetwopen-e2334757-s001.pdf]

Data Sharing Statement

Penz1. Colorectal Cancer and Precursor Lesion Prevalence in Adults Without Symptoms Aged <50 Years in Austria. JAMA Netw Open. Published online December 6, 2023. doi:10.1001/jamanetworkopen.2023.34757

Data
Data available: No
